# Cost-Effectiveness of Clinical Decision Support System in Improving Maternal Health Care in Ghana

**DOI:** 10.1371/journal.pone.0125920

**Published:** 2015-05-14

**Authors:** Maxwell Ayindenaba Dalaba, Patricia Akweongo, Raymond Akawire Aborigo, Happiness Pius Saronga, John Williams, Antje Blank, Jens Kaltschmidt, Rainer Sauerborn, Svetla Loukanova

**Affiliations:** 1 University of Heidelberg, Institute of Public Health, Heidelberg, Germany; 2 Navrongo Health Research Centre, Navrongo, Ghana; 3 University of Ghana, School of Public Health, Accra, Ghana; 4 Muhimbili University of Health and Allied Sciences, Behavioural Sciences Department, School of Public Health and Social Sciences, Dar es Salaam, Tanzania; 5 Department of Clinical Pharmacology and Pharmacoepidemiology, University Hospital of Heidelberg, Heidelberg, Germany; Institute of Endocrinology and Metabolism, IRAN, ISLAMIC REPUBLIC OF

## Abstract

**Objective:**

This paper investigated the cost-effectiveness of a computer-assisted Clinical Decision Support System (CDSS) in the identification of maternal complications in Ghana.

**Methods:**

A cost-effectiveness analysis was performed in a before- and after-intervention study. Analysis was conducted from the provider’s perspective. The intervention area was the Kassena- Nankana district where computer-assisted CDSS was used by midwives in maternal care in six selected health centres. Six selected health centers in the Builsa district served as the non-intervention group, where the normal Ghana Health Service activities were being carried out.

**Results:**

Computer-assisted CDSS increased the detection of pregnancy complications during antenatal care (ANC) in the intervention health centres (before-intervention= 9 /1,000 ANC attendance; after-intervention= 12/1,000 ANC attendance; P-value=0.010). In the intervention health centres, there was a decrease in the number of complications during labour by 1.1%, though the difference was not statistically significant (before-intervention =107/1,000 labour clients; after-intervention= 96/1,000 labour clients; P-value=0.305). Also, at the intervention health centres, the average cost per pregnancy complication detected during ANC (cost –effectiveness ratio) decreased from US$17,017.58 (before-intervention) to US$15,207.5 (after-intervention). Incremental cost –effectiveness ratio (ICER) was estimated at US$1,142. Considering only additional costs (cost of computer-assisted CDSS), cost per pregnancy complication detected was US$285.

**Conclusions:**

Computer –assisted CDSS has the potential to identify complications during pregnancy and marginal reduction in labour complications. Implementing computer-assisted CDSS is more costly but more effective in the detection of pregnancy complications compared to routine maternal care, hence making the decision to implement CDSS very complex. Policy makers should however be guided by whether the additional benefit is worth the additional cost.

## Introduction

In Ghana, despite the fact that maternal health services are free to women in all public health facilities, maternal deaths are still unacceptably high due to other contextual factors. The maternal mortality ratio (MMR) in Ghana is estimated at 350 maternal deaths per 100,000 live births [1]. Near-miss cases, defined as women who experience and survive a severe health condition during pregnancy, childbirth or postpartum, occur more commonly than maternal deaths [2–4]. The incidence of maternal near-misses in Ghana was estimated at 28.6 cases per 1,000 live births[5].

Most maternal complications and deaths occur as a result of insufficient quality of care during pregnancy and labour. Efforts towards reducing maternal morbidity and mortality in low-income countries such as Ghana have focused on improving the quality of antenatal care (ANC) and delivery services. The recommended intervention to improve quality of ANC and delivery care is the use of standard guidelines such as the WHO guide for “Pregnancy, childbirth, postpartum and newborn care (PCPNC)”, which is the source for national guidelines for most African countries including Ghana. These guidelines which are predominantly paper-based have been reported to be poorly used by health workers [6].

In recent times, computer-assisted Clinical Decision Support Systems (CDSS) have been used to facilitate the application of knowledge at the point of care. The system simplifies adherence to guidelines leading to improved performance [7–13]. Computer-assisted CDSS uses the characteristics of individual patients to match a computerized knowledge base for software algorithms to generate patient specific recommendations at the point of care [9]. Since the introduction of computer-assisted CDSS to improve health care delivery, no study has evaluated its cost-effectiveness for ANC and delivery services in primary care facilities in rural Africa. The computer-assisted CDSS has financial implications as it involves additional cost such as buying of computers, training of health workers, costs of monitoring/supervision, among others. Resources for health care are however limited, thus necessitating informed choices among the range of alternatives available to health systems.

The Navrongo Health Research Centre QUALMAT (Quality of maternal and neonatal care) project implemented a computer-assisted Clinical Decision Support System (CDSS) in six health care centres in the Kassena-Nankana district (KND) of Ghana. The computer-assisted CDSS aimed to enhance health workers adherence to maternal health guidelines, to identify complications and make accurate decisions such as referrals and ultimately reduce maternal morbidity and mortality. This paper examined whether the computer-assisted CDSS is an attractive economic option in the KND in northern Ghana. We hypothesised that the use of computer-assisted CDSS will be cost-effective in the detection of pregnancy complications and in the reduction of labour complications when compared with usual manual pattern of maternal care.

## Materials and Methods

### Ethical statement

The study was part of the Quality of maternal and neonatal care (QUALMAT) project, which aimed to improve the quality of maternal and prenatal care in Ghana by testing two interventions—a computer-assisted clinical decision support system and performance-based incentives. The study was approved by the ethics committee of the University of Heidelberg (S-173/2008) and the Institutional Ethical Review Board of the Navrongo Health Research Centre in Ghana (NHRCIRB 085).

### Study area

The study was carried out in the Kassena-Nankana East and West districts as well as the Builsa district in northern Ghana. In this study, the Kassena-Nankana East and West districts would be referred to by their previous name—the Kassena-Nankana District (KND). The KND occupies a total area of about 1,675 square kilometres with a population of approximately 152,000 [14]. Maternal mortality ratio was estimated at 637 per 100,000 live births for the period 1995–1996. However, for the period 2002–2004, MMR declined to 373 maternal deaths per 100,000 live births, representing a 40% reduction in the ratio [15]. In 2010, the MMR in of the KND was 367 maternal deaths per 100,000 live births [16].

The KND has one hospital located in the capital town Navrongo that serves as a referral point for all health facilities in the district. There are six main health centres, one private clinic and twenty seven Community-based Health Planning and Services (CHPS) compounds. CHPS is a health care strategy that places a Community Health Officer (CHO) in the community to provide health care services [17].

The Builsa district that served as the non-intervention district lies southwest of the KND and covers an area of 2,220 square kilometres with a population of approximately 95,800. It has a hospital located in the capital town Sandema that serves as a referral point for all health facilities in the district. There are six main health centres and thirteen CHPS compounds. In 2010, the district recorded a MMR of 259 maternal deaths per 100,000 live births [18].

In both the Kassena-Nankana and Builsa districts, subsistence agriculture is the mainstay of the people. The districts are in one of the poorest regions in Ghana with poverty incidence of 88% [19,20].

### Maternal care provision

In Ghana, health service delivery is organized in five levels: tertiary, regional, district, sub-district (health centres and clinics), and community (CHPS) level. The health centres included in this study fall under the sub-district level and provide outpatient services, antenatal care and deliveries. All maternal health services, including antenatal care, spontaneous and assisted delivery care, caesarean section, management of emergency obstetric conditions, and postnatal care are provided at the health centres at no cost to women [21].

The current maternal health care protocol being used at the health centres is the “National Safe Motherhood Service Protocol (2008)” which is based on the WHO PCPNC guide for essential practice [22]. The protocol outlines step-by-step actions for comprehensive maternal care, identification and treatment of common pregnancy-related complications and when to refer clients. This reference guide aims to ensure that health workers know what is expected of them when providing maternal and newborn services. It is recommended that pregnant women without danger signs of complication should make a minimum of four visits. More frequent visits are recommended for those with complications.

As part of ANC, health workers take comprehensive history of clients. This includes age, parity, gestational age, general wellbeing, gastro-intestinal symptoms, genital symptoms and so on. Measurements such as weight, height, blood pressure, heart rate and temperature are routinely done and recorded. Basic laboratory tests such as HIV, protein and glucose are also conducted. All pregnant women are counselled on maternal danger signs and preventive behaviours. The women are also given iron, mebendazole, Sulphadoxine-Pyrimethamine to prevent anaemia, intestinal worm infestation, and malaria infections respectively.

### Study design

**Fig 1:**
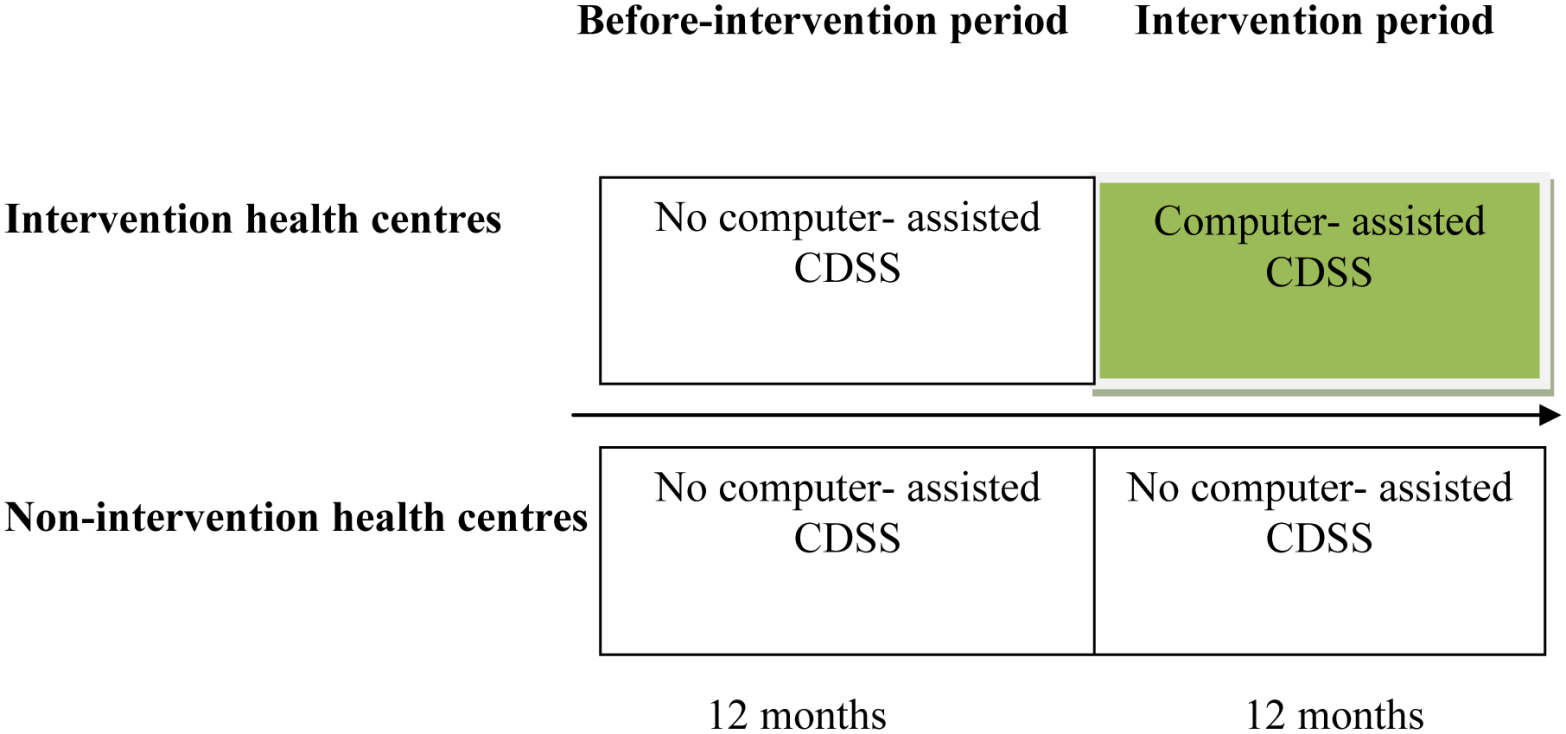
Study design.

A cost-effectiveness analysis was performed within a before- and after-intervention study with a non-intervention group (Fig 1). Analysis was conducted from the provider’s perspective. The KND was the intervention district where computer-assisted CDSS was implemented in 6 health centres for midwives to use in maternal health care. The Builsa district served as the non-intervention area where the normal Ghana Health Service (GHS) activities were being carried out in the six health centres.

### Description of the intervention

Computer-assisted Clinical Decision Support System (CDSS) was implemented in April, 2012 in all six health centres in the KND. Each health centre received a laptop with the CDSS software installed on it. One midwife and one community health nurse from each health centre were selected and trained to use the computer-assisted CDSS during antenatal and delivery care. Activities conducted by the nurses using the computer-assisted CDSS are not different from the paper based guideline approach as explained above. When a woman arrives at the health centre for ANC, the midwife (CDSS user) records all vital information of the woman into the computer-assisted CDSS by following step-by step activities. These activities or checks include questions on medical history of the woman, clinical and physical examinations and the provision of counselling and preventive measures. For instance, the system would request that the health worker takes blood pressure (BP). If two readings of diastolic BP are equal or greater than 90mmHg, the system prompts the health worker to look for oedema of feet, hands, face and ankles. The system also request for the check of severe headaches, blurred vision or epigastric pain. If any of these signs are present, the system suggests hypertensive disorders or mild-pre-eclampsia and would recommend referral. Also during labour, the computer-assisted CDSS is used to monitor labour by charting a partograph. This involves the recording of patient information, fetal heart rate, amniotic fluid, moulding, cervical dilatation, alert line etc. If dilatation crosses the “action” line, the system recommends referral.

Generally, the computer-assisted CDSS prompts health workers to provide complete care and then gives recommendations or alerts the health worker when there are danger signs. Any woman with danger signs that cannot be managed at the health centre is referred to a hospital. Detailed description of the implementation of the computer-assisted CDSS is presented elsewhere [23].

### Source of data and analysis

#### Health centre cost

Health centre cost data was collected between July and December 2010 in all the 12 health centres. All health-care related costs for the year 2010 were collected from a provider’s perspective. Data were collected through interviews with health centre staff, document review, physical measuring of rooms and counts of equipment. The cost collected included recurrent costs such as personnel, administration, pharmacy, vaccines, laboratory, medical supplies and capital costs such as building, vehicle and equipment costs. Recurrent costs are items that are used up during a year and are usually purchased regularly whiles capital costs are items with a lifespan greater than one year. The step-down allocation (SDA) approach was used for the analysis. Total health facility cost as well as costs associated with ANC and delivery services were calculated. Detailed description of the cost data collection, analysis and results have been reported elsewhere [24].

#### Cost of computer-assisted CDSS

All the activities and associated cost that occurred during the computer-assisted CDSS implementation period (October 2009 through April 2013) were included in the cost estimation. Thus, costs of personnel, trainings, meetings, transportation, overheads and equipment were collected and summed. Detailed description of costs of implementation of the computer-assisted CDSS have been reported in earlier publication [23].

#### Effectiveness data

Though maternal mortality ratios are high in many sub-Saharan Africa countries, the actual number of deaths in a health facility is not usually adequate to assess impact of maternal health interventions. Maternal mortality ratios alone cannot be used to measure change over short periods of time [25]. Maternal morbidity such as pregnancy complications or near miss is now being used as an alternative outcome measure to evaluate and improve quality of care [4,26].

For this reason, our main effectiveness or outcome measure was identified maternal complications. Maternal complications were defined in this study as women who were diagnosed by health workers during ANC consultations or during labour to have complications and were referred from the study health centres to the next level of care (hospitals) for treatment due to limitations within the health centres.

The before-intervention study captured 12 months (April 2011 to March 2012) of data on routine services for maternal health before the implementation of the computer-assisted CDSS. The after-intervention study captured 12 months (April 2012 to March 2013) of data after computer-assisted CDSS implementation. Data were collected for before and after computer-assisted CDSS implementation in both the intervention and non-intervention health centres. We reviewed registers from the health centres and collected data on the number of antenatal consultations, labour cases, complications detected during ANC and labour.

We calculated the number of complications that were detected during antenatal care and labour at the health centres at the before- and after-intervention periods. The number of complications detected during ANC visits was calculated as the number of complications detected during ANC consultations per 1,000 ANC consultations. Also, the number of complications detected during labour was calculated as the number of detected complications during labour per 1,000 labour clients. The difference in proportions between the before-and-after interventions was tested. P-values of < 0.05 were considered statistically significant.

### Cost effectiveness analysis (CEA)

In the performance of CEA, the incremental cost—effectiveness ratio (ICER), is commonly used [27]. The ICER represents the additional cost of one unit of outcome gained by one strategy compared with another. The ICER is expressed as the ratio of the difference in cost to the difference in effect between the two comparators. See formula below:

$$ICER\;=\;\frac{\Delta C}{\Delta E}\;=\;\frac{\left(C_{B}-C_{A}\right)}{\left(E_{B}-E_{A}\right)}$$

Where C_A_, E_A_ represent cost and effects of intervention A (intervention currently in place) respectively;

and C_B_, E_B_ represent cost and effects of intervention B (new intervention under consideration for adoption) respectively.

In this study, the new intervention (B) was the implementation of the computer-assisted CDSS in the health centres and the current intervention (A) was the routine provision of MNC care in the health centres.

C_A_ was the health centre cost on ANC/delivery services before CDSS intervention,

E_A_ was complications detected during ANC/delivery before CDSS intervention.

C_B_ was health centre cost on ANC/delivery services after CDSS intervention (i.e. health centre cost on ANC/delivery services plus CDSS intervention cost).

E_B_ was complications detected after CDSS intervention.

In addition, incremental cost, incremental effect and cost effectiveness ratios were calculated. Incremental cost was calculated as health centre cost on ANC and delivery services after CDSS intervention cost minus health centre cost on ANC and delivery services before CDSS intervention (C_B—_C_A_). Incremental effect was calculated as complications detected after CDSS intervention minus complications detected during ANC and delivery before CDSS intervention (E_B—_E_A_). Cost-effective ratio was calculated by dividing incremental cost by the incremental effect.

## Results

### ANC and labour complications

As shown in Table 1, there was a statistically significant increase in the number of complications detected (conditions such as pre-eclampsia/hypertension, haemorrhage and infections/sepsis) during ANC consultations in the intervention health centres after computer-assisted CDSS implementation. Thus in the intervention health centres, an average of 3 cases per 1,000 ANC attendances were detected (before-intervention = 9/1,000 ANC attendance; and after-intervention = 12/1,000 ANC attendance; P-value = 0.010). In the non-intervention health centres, the average number of pregnancy complication detected after the CDSS intervention period did not change (before = 16/1,000 ANC attendance; after = 16/1,000 ANC attendance; P-value = 0.963).

**Table 1:** Pregnancy complications.

	Before- intervention (Baseline)	After-intervention	Difference (After minus before)	P-value
*ANC*	*Average complications detected (per 1*,*000 ANC attendance)*	*Average complications detected (per 1*,*000 ANC attendance)*		
Intervention	9	12	3	0.01*
Non-intervention	16	16	0	0.963
Difference (Intervention—Non-intervention)	-7	-4	3	
P-value	0.001*	0.014*		
*Delivery*	*Average complications detected (per 1*,*000 labour cases)*	*Average complications detected (per 1*,*000 labour cases)*		
Intervention	107	96	11	0.305
Non-intervention	133	100	33	0.009*
Difference (Intervention—Non- intervention)	-26	-4	-22	
P-value	0.033*	0.7297		

*P<0.05 (5% level of significance)

In the intervention health centres though there was a decrease in the proportion of labour complications detected after CDSS intervention, it was not statistically significant (before = 107/1,000 labour cases; after = 97/1,000 labour cases; P-value = 0.304). However, there was a significant decrease in the number of labour complications after the CDSS intervention period in the non-intervention health centres (before = 133/1,000 labour cases; after = 100/1,000 labour cases; P-value = 0.009) (Table 1).

### Cost-effectiveness analysis (CEA)

As presented in Table 1, at the intervention health centres, there was no statistically significant difference in the decrease in complications during labour after the computer-assisted CDSS intervention. In addition, there was no statistically significant difference in the number of labour complications detected between the intervention and the non-intervention health centres. Therefore, cost-effectiveness analysis was not performed on complications detected during labour. Cost-effectiveness analysis was only performed on complications detected during ANC consultations at the intervention health centres.

As shown in Table 2, before CDSS intervention, the average health facility cost on ANC services in the six intervention health centres was US$ 14,975.47 [24]. The average cost of computer-assisted CDSS implementation was estimated at US$3,425.60 [23]. Assuming that the CDSS was used for only ANC services, then the average health facility cost of ANC services after CDSS intervention will increase to US$18,401 (US$ 14,975.47 plus US$3,425.60). Thus, before CDSS intervention, an average amount of US$14,976 was spent at the health centres for the provision of ANC services and the average number of complications detected was 9 per 1, 000 ANC attendances. The introduction of computer-assisted CDSS increased the cost to US$18,401 and the number of complications detected increased to 12 per 1, 000 ANC attendance. The cost per pregnancy complication detected during ANC (cost—effectiveness ratio) decreased from US$1,664 (before-intervention) to US$1,533 (after-intervention), representing 9% cost reduction. The incremental cost—effectiveness ratio (ICER) was estimated at US$1,142. Thus it would require an extra US$1,142 to detect one pregnancy complication using computer-assisted CDSS compared to the routine method. Considering only cost of computer-assisted CDSS (US$3,425) implementation, the cost per pregnancy complication detected was estimated at US$285.

**Table 2:** Cost-effectiveness analysis.

Intervention	Cost (Average ANC services cost)	Incremental cost (AI cost—BI cost)	Effect (Average complication detected per 1000 ANC attendance)	Incremental effect per 1000 ANC attendance (AI effect—BI effect)	Average cost effectiveness ratio (Cost/Effect)	Incremental cost effectiveness ratio_ICER (Incremental cost/incremental effect)
Before-Intervention (BI)	14,975		9		1,664	
After—Intervention (AI)	18,401	3,426	12	3	1,533	1,142

### Sensitivity analysis

One- way sensitivity analysis was conducted to determine whether changes to variables such as discount rate and life expectancy of capital costs will significantly change the results. As presented in the sensitivity analysis table (Table 3), varying the discount rate and life expectancy of capital cost had less than 6% change in ICER. However, given that the effect is the denominator, when the identified number of maternal complications (effect) increases, the ICER will reduce.

**Table 3:** Sensitivity analysis.

Intervention /variables	Average ANC cost	Incremental cost	Average complications (per 1000 ANC attendance)	Incremental effect (per 1000 ANC attendance)	Cost effectiveness ratio (Cost/Effect)	Incremental cost effectiveness ratio (ICER)
*3% Discount rate*						
Before-intervention	14975		9		1664	
After-intervention	18401	3426	12	3	1533	1142
*5% discount rate*						
Before-intervention	15049		9		1672	
After—intervention	18495	3446	12	3	1541	1149
*20 years Life expectancy for building*						
Before-intervention	14981		9		1665	
After-intervention	18407	3426	12	3	1534	1142
*10 years Life expectancy for computers*						
Before-intervention	14975		9		1664	
After-intervention	18241	3266	12	3	6080	1089

## Discussion

It is important to identify and treat danger signs, as they are likely to cause life-threatening complications or death. In the intervention health centres, we observed an increase in the detection of pregnancy complications during the provision of ANC after computer-assisted CDSS intervention. There was a decrease in the number of complications during labour in the intervention health centres, though the decrease observed was not statistically significant. Even though the decrease in labour complications was not statistically significant, it is important to note that given the consequences of complications during labour, any marginal decrease is important. Also, the computer-assisted CDSS was evaluated over a one-year period and there is the possibility of a significant reduction of delivery complications when the system is used for a longer period.

Although the use of a partograph is a well-known intervention to detect and manage labour, it is often not used or not used correctly by midwives [28]. Given that the computer-assisted CDSS facilitates the use of a partograph, the reduction in labour complications could be attributed to the CDSS. The reduction in the number of complications during labour could also be due to the increase in the number of pregnancy complications detected and managed during the ANC visits. Usually women identified during ANC to have complications are advised to deliver at the hospital.

Unexpectedly, at the non-intervention health centres where CDSS was not used in the management of pregnant women, there was a significant reduction in the number of delivery complications. This could be due to the presence of other maternal health intervention activities within the control district. For instance, during the study period, the Ghana Essential Health Project (GEHIP), which aimed to improve the quality of maternal and neonatal health instituted procedures for promoting facility-based delivery as well as training on emergency referral systems to prevent delay in care when emergencies arise [29].

Overall, it is more costly but more effective to add computer-assisted CDSS to the conventional method in the detection of pregnancy complications during ANC. Considering average cost per compilations (cost effectiveness ratio), about 9% cost savings were made and 3 more complications detected comparing the use of computer-assisted CDSS to the conventional method. The implementation of computer-assisted CDSS will now depend on whether the additional benefit is worth the additional cost. There are no firm benchmarks or thresholds to determine whether an intervention is cost-effective or not [30]. However, the Commission on Macroeconomics and Health and WHO suggested that interventions with ICER that is less than the per capita GDP is “very cost-effective” [31–34]. Therefore given that the ICER estimated in our study (US$1,142) is lower than the GDP per capita in Ghana (US$1,850 in 2013), computer-assisted CDSS can be considered as cost-effective. Decision makers will also have to consider the burden of maternal morbidity and mortality to households and the economy to decide whether the computer-assisted CDSS is cost-effective or not. Maternal morbidity and mortality can endanger the survival and wellbeing of families [35–38]. Households in the study setting spend about 3% of their annual household expenditure on payments for maternal complications and face the risk of incurring catastrophic health expenditure[39]. Studies in other parts of Africa have reported that one maternal mortality reduces per capita GDP by US$0.36 per year [40]. This suggest that any additional investment in the use of computer-assisted CDSS, that leads to early detection of complications will be useful in averting deaths and reducing household costs associated with life-threatening complications. Such investment is not only fair for gender equity but also appropriate to improve economic growth and development.

### Limitations of the study

The period for the evaluation was one year and could have accounted for the lower effects. We are uncertain whether the full effect of the system has been realized. Also, we were not able to make a meaningful comparative analysis between the intervention and non-intervention health centres because there were other related maternal health intervention activities that took place at the control health centres around the same time period.

## Conclusion

Computer-assisted CDSS can enable midwives to identify complications (danger signs) during ANC consultations and facilitate marginal reduction in delivery complications. The system is more costly but more effective in the detection of pregnancy complications compared to the routine method. Hence the decision to implement computer-assisted CDSS is not straightforward. An additional cost of US$1,142 (ICER) would be required to identify one additional pregnancy complication case. The implementation of computer-assisted CDSS will now depend on whether the additional benefit is worth the additional cost. Health systems would therefore need to consider the short- and long- term benefits and health budget impact before rolling out computer-assisted CDSS. Although this study has provided useful evidence on computer-assisted CDSS in the intervention district, it would be important to know if these results are applicable to the rest of the country. Further studies with longer duration and in different settings are therefore needed before rolling out computer-assisted CDSS.
